# Rapid Separation of Asiatic Acid, Quercetin, and Kaempferol from Traditional Chinese Medicine *Centella asiatica* (L.) Urban Using HSCCC-Semi-Prep-HPLC and the Assessment of Their Potential as Fatty Acid Synthase Inhibitors

**DOI:** 10.1155/2023/7769368

**Published:** 2023-10-10

**Authors:** Binbin Xia, Yali Li, Yang Liu, Wenfang Sun, Jing Chen, Liushui Li, Jingyao Pang, Xianjun Liu, Shicai Chen, Hua Cheng

**Affiliations:** Department of Pharmacy, Beijing Luhe Hospital Affiliated to Capital Medical University, Beijing, China

## Abstract

The main objective of this study was to rapidly separate asiatic acid (AA), quercetin (QCN), and kaempferol (KPL) from *Centella asiatica* (L.) Urban using high-speed counter-current chromatography (HSCCC) in tandem with the UV detector of semipreparative high-performance liquid chromatography (Semi-Prep-HPLC) and to evaluate their potential as inhibitors of fatty acid synthetase (FAS). To efficiently prepare large amounts of AA, QCN, and KPL from *Centella asiatica* (L.) Urban, rapid and simple methods by HSCCC were established respectively based on the partition coefficients (*K* values) of crude samples. The conditions of HSCCC-Semi-Prep-HPLC for the large-scale separation of AA, QCN, and KPL from *Centella asiatica* (L.) Urban were established and optimized. This included selecting the solvent system, flow rate, rotation speed, and so on. HSCCC-Semi-Prep-HPLC was successfully applied to separate and purify AA, QCN, and KPL, with *n*-hexane-*n*-butanol-methanol-water (3 : 1 : 3 : 3, V : V : V : V) as the solvent system for AA, which was detected at a wavelength of 210 nm with the stationary phase retention of 70%, and with *n*-hexane-ethyl acetate-methanol-water (0.8 : 0.9 : 1.2 : 1, V : V : V : V) as the solvent system for the co-separation of QCN and KPL, which was detected at a wavelength of 254 nm with the stationary phase retention of 65%. AA could be isolated at a large scale with high purity (>91.0%) in only one-step HSCCC-Semi-Prep-HPLC separation (within 150 min) under the optimized conditions. Meanwhile, QCN and KPL could be simultaneously isolated at a large scale with high purity (>99.1%) by another one-step HSCCC-Semi-Prep-HPLC separation (within 240 min) under the optimized conditions. The assessment of inhibition potential revealed that AA exhibited the strongest inhibitory effect on FAS, with an IC_50_ of 9.52 ± 0.76 *μ*g/mL. Madecassic acid (MA) followed closely with IC_50_ values of 10.84 ± 0.92 *μ*g/mL. QCN and KPL showed similar and relatively weaker inhibitory effects on FAS, with IC_50_ values of 43.09 ± 2.98 *μ*g/mL and 36.90 ± 1.83 *μ*g/mL, respectively. Overall, the HSCCC-Semi-Prep-HPLC method proved to be a highly efficient and reliable technique for separating AA, QCN, and KPL from *Centella asiatica* (L.) Urban, and the isolated compounds showed potential as FAS inhibitors.

## 1. Introduction

*Centella asiatica* (L.) Urban, also known as Gotu Kola [1], is a traditional Chinese medicine that has been recorded as a medicinal herb in Shennong's Classic of Materia Medica more than 2000 years ago. According to the 2020 edition of the Chinese Pharmacopoeia, *Centella asiatica* is cold in nature and has a bitter and pungent taste, with the functions of clearing heat and dampness, detoxification, and reducing swelling. It is commonly used in the treatment of damp-heat jaundice, heatstroke, diarrhea, hematuria and dysuria, abscesses and sores, bruises and sprains, infectious hepatitis, skin diseases, epidemic cerebrospinal meningitis, etc. [2, 3]. Asiatic acid (AA), the aglycones of ursane-type pentacyclic triterpenoids, is the major active component that was isolated and identified from *Centella asiatica* (L.) Urban, and our previous research has shown that *Centella asiatica* (L.) Urban is rich in AA, with a content of 1.115 g·kg^−1^ [3, 4]. Quercetin (QCN) and kaempferol (KPL), representative of the flavonoids, are also the main components of *Centella asiatica* (L.) Urban, with the content of >1.28 g·kg^−1^ and >7.82 g·kg^−1^, respectively [5]. AA has been found to have various pharmacological effects, such as promoting the healing of skin wounds [6], anti-inflammatory [7, 8], antioxidant [9, 10], antidepressant [11], anti-Alzheimer's disease [12, 13], antitumor [14, 15], nerve repair [16, 17], antiobesity [18, 19], and protection of cardiovascular and cerebrovascular systems [20, 21]. QCN and KPL have been shown to have antioxidant [22, 23], anti-inflammatory [22, 24], antihypertensive, cardioprotective [24, 25] effects.

To further explore the pharmacological effects and research mechanisms of AA, QCN, and KPL, there is a need to isolate these compounds. However, the availability of the reports on the isolation of these compounds from the *Centella asiatica* (L.) Urban is , at present, very scarce. Our research group has previously reported the isolation and purification of AA from *Centella asiatica* (L.) Urban using silica gel column chromatography [26]. However, this method is found to be tedious and time-consuming for the isolation and purification of AA from *Centella asiatica* (L.) Urban. Moreover, when the AA extract was subjected to three rounds of separation and purification using silica gel column chromatography, we were only able to achieve a yield of 79.0% [26]. Additionally, this method has the drawback of compound loss due to the highly irreversible adsorptive property of the solid matrix [27]. High-speed countercurrent chromatography (HSCCC) is an advanced liquid-liquid partition chromatography technology that has been widely used for the separation and purification of various natural products due to its several advantages [27]. One of its well-known benefits is that it does not require a solid support matrix for the stationary phase, allowing the preparative separation of solutes in a two-phase solvent system [28]. This feature eliminates the irreversible adsorption loss of the sample caused by the solid support matrix in traditional chromatography columns [29]. In addition, HSCCC offers several advantages, such as high recovery, high efficiency, simple pretreatment operation, high reproducibility, and ease of scaleup [27–29]. However, selecting an appropriate solvent system for separation is a critical operation.

To date, as far as we know, no report has been reported on the use of HSCCC for the separation and purification of QCN and KPL from *Centella asiatica* (L.) Urban. Du et al. [30] described the separation of four ursane triterpenoids (containing AA) from a crude extract of *Centella asiatica* using HSCCC. The separation was achieved by employing a solvent system consisting of a three-step gradient with a time-dependent increase in the eluting strength of the mobile phase, which involved four solvent systems [30]. In this experiment, the lower phase of the solvent system composed of *n*-hexane/*n*-butanol/0.05M NaOH (5/1/6, V/V/V) served as the stationary phase, while the upper phase was used as the initial mobile phase [30]. The flow rates were significantly reduced from the initial 5.0 mL/min to 3.0 mL/min (step 1), 2.0 mL/min (step 2), and 1.5 mL/min (step 3), which was accompanied by a stepwise increase in the *n*-butanol content in the mobile phase, starting from a ratio of 5 : 1 and progressing to 1 : 1 (step 1), 1 : 2 (step 2), and 1 : 4 (step 3) of *n*-hexane/*n*-butanol [30]. Thin-layer chromatography (TLC) with an ethyl acetate-methanol-water (8 : 2 : 1, V/V/V) solvent system was employed to evaluate the HSCCC fractions. The triterpenoids were visualized by spraying with 3% sulfuric acid in ethanol and heating to 110°C for 5 minutes on a hot plate [30].

However, this HSCCC method had several limitations. It required manual changes of solvent systems and adjustments of flow rates, making it relatively cumbersome and time-consuming. Real-time online monitoring was not feasible, and TLC was used for triterpenoid detection instead. Furthermore, the study did not measure the distribution coefficients of the samples in their respective solvent systems, did not screen for the optimal solvent system, and did not report the purity of the samples obtained after a single HSCCC separation. The lack of real-time tracking for the distillate represented a significant limitation. Therefore, there is a need to develop a simple, rapid, and real-time trackable HSCCC-Semi-Prep-HPLC method to achieve fast separation of AA.

**Figure 1:**
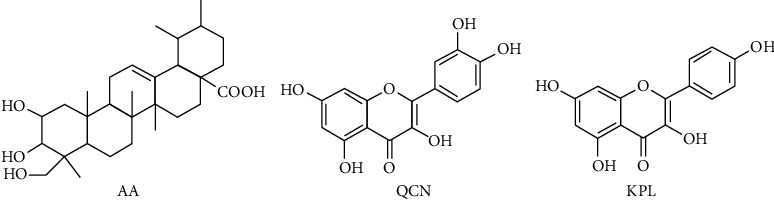
Chemical structure of asiatic acid (AA), quercetin (QCN), and kaempferol (KPL).

The purpose of this work, therefore, was to establish an effective and convenient HSCCC method for preparative isolation and purification of the target compound AA, QCN, and KPL directly from the crude extract of *Centella asiatica* (L.) Urban. Furthermore, potential inhibiting effects of these ingredients on fatty acid synthetase (FAS) were evaluated by the respective FAS inhibitory test. The chemical structures of AA, QCN, and KPL are shown in Figure 1.

## 2. Materials and Methods

### 2.1. Materials and Reagents

*Centella asiatica* (L.) Urban was purchased from Tong Ren Tang Pharmacy in Beijing. Acetyl CoA, malonyl CoA, NADPH, and reference substance of AA (purity >95%) were purchased from Sigma-Aldrich. The reference substance of QCN (purity >98%), KPL (purity >98%), and madecassic acid (MA, purity >97%) were purchased from Tauto Biotech, Shanghai, China. All organic solvents used for the preparation of enriched extract and for HSCCC separation were of analytical grade (Beijing Chemical Works, Beijing, China). The methanol and acetonitrile used for HPLC were of chromatographic grade (Thermo Fisher Scientific, Waltham, MA, USA), and the water used was deionized. Fatty acid synthetase was isolated and purified from fresh duck liver that had been prepared and stored according to the method reported [31] and was identified as a single band by polyacrylamide gel electrophoresis (SDS-PAGE). The high-speed frozen centrifuge used in this manuscript is the MIKRO-22R (Hettich GmbH, Germany).

### 2.2. Apparatus

The HSCCC equipment utilized in this paper was a TBE-300C HSCCC system (Tauto Biotech, Shanghai, China), consisting of three multilayer coil separation columns connected in series (inner diameter of tubing = 1.6 mm, total volume = 300 mL, *β*‐values = 0.5–0.8), and a 20 mL sample loop. The instrument's revolution speed can be regulated using a speed controller within the range of 0–1000 rpm. The HSCCC system was equipped with a TBP-5002 constant-flow pump (Tauto Biotech, Shanghai, China), a HX-1050 thermostatic circulating instrument (BIOCOOL, Beijing, China), and a 1525 semipreparative high-performance liquid chromatography system (which included a binary gradient pump, UV-2487 dual-wavelength UV detector, empower workstation, which were all manufactured by Waters Corporation, USA). Additionally, a Waters 1525 series analytical HPLC system (which included a binary gradient pump, UV-2487 UV detector, online vacuum degasser, and automatic sampler) was used for analytical determination. The UV-2550 UV-visible spectrophotometer (Shimazu Corporation, Japan), the LABOROTA type 4000 rotary evaporator (Heidolhp, Germany), the SHZ-III circulating water vacuum pump (Yarong Biochem, Shanghai, China), the KQ-250DB type CNC ultrasonic cleaner, and the Mill (Tasite Instrument, Tianjin, China) were also used in this paper. The ESI-MS^n^ system, controlled by Xcalibur® software (version 1.3), consisted of a Finnigan LCQ Deca XP ion-trap spectrometer equipped with an electrospray source (Thermo Finnigan, San Jose, CA, USA).

### 2.3. Methods

#### 2.3.1. Chromatographic Conditions for the Determination of AA

**Figure 2:**
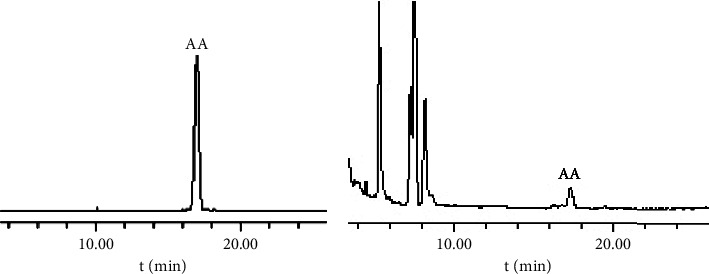
HPLC chromatograms of the AA reference substance and the extract B from *Centella asiatica* (L.) Urban at 210 nm [26].

Based on the chromatographic conditions established earlier by our group, the chromatographic column used was Symmetry C18 (4.6 mm × 250 mm, 5 *μ*m, Waters, USA) with acetonitrile-10 mmol/L ammonium acetate aqueous solution (19 : 31, v/v) as the mobile phase. The flow rate was 1.0 mL/min, the detection wavelength was set to 210 nm, the column temperature was maintained at 25°C, and the sample size was 20 *μ*L.  Figure 2 displays the chromatogram of the AA reference substance (purity >95%).

To determine the most suitable mobile phase, various ion-pairing reagents were added to improve the resolution of the AA peak and impurity peak in the HPLC chromatogram of the *Centella asiatica* (L.) Urban extract. Two binary solvent systems, methanol-water and acetonitrile-water, were used as mobile phases. Our team previously investigated the effect of methanol or acetonitrile as organic phases on the separation and analysis of AA. We found that methanol has significant end absorption at 210 nm, while acetonitrile does not. Therefore, acetonitrile was chosen as the organic phase.

Several mobile phases, including acetonitrile-acetic acid aqueous solution, acetonitrile-formic acid aqueous solution, acetonitrile-phosphate buffer solution, acetonitrile-ammonium chloride aqueous solution, and acetonitrile-*β*-cyclodextrin aqueous solution, were screened for their effects on the HPLC chromatograms of the *Centella asiatica* (L.) Urban extract. The proportions of the mobile phases and their solution pH (pH = 3–6) were further adjusted for analysis. However, none of the above mobile phases effectively separated AA and the separation degree between the AA peak and impurity peak was <1.5.

**Figure 3:**
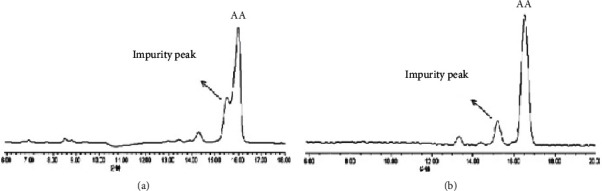
HPLC chromatograms of the sample before (a) and after (b) optimization.

We then used acetonitrile-10 mmol/L ammonium acetate aqueous solution (19 : 31, V/V), which resulted in a good separation effect. The separation degree between the AA peak and impurity peak was >2, with a good peak shape and no front or tailing peak, and an AA retention time of about 17 min. Therefore, the acetonitrile-10 mmol/L ammonium acetate aqueous solution (19 : 31, V/V) was chosen as the mobile phase for qualitative and quantitative analyses of AA in the *Centella asiatica* (L.) Urban extract.  Figure 3 shows the HPLC chromatograms of the AA-containing samples before and after optimizing the chromatographic conditions.

In addition, we investigated the effects of flow rate and column temperature on the retention time and separation degree of AA. We found that both flow rate and column temperature significantly affected the retention time of AA and had a certain effect on the separation degree between AA and impurities. When the flow rate and column temperature were low, the retention time of the sample significantly increased, and the separation effect was better. Considering the effects of retention time and separation degree, a flow rate of 1.0 mL/min and a column temperature of 25°C were ultimately chosen.

#### 2.3.2. Chromatographic Condition for the Determination of QCN and KPL

**Figure 4:**
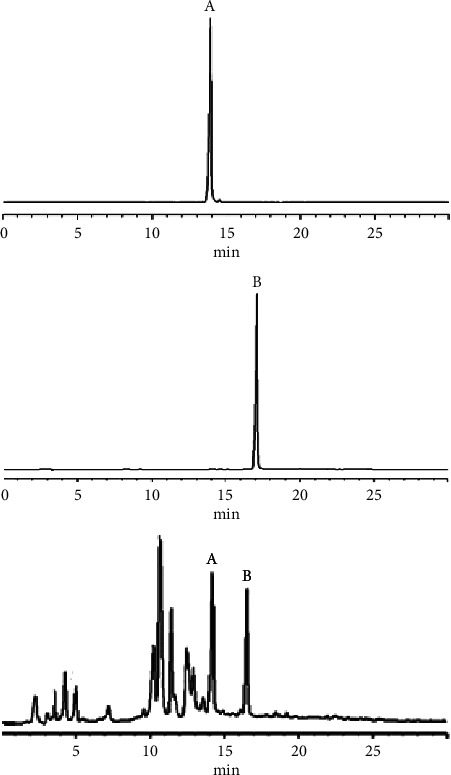
HPLC chromatogram of QCN (A) and KPL (B) and crude extract A from *Centella asiatica* (L.) Urban at 254 nm, respectively.

The chromatographic column used in the study was also Symmetry C18 (4.6 mm × 250 mm, 5 *μ*m, Waters). A binary linear gradient elution method was employed for the mobile phase, with acetonitrile as phase A and 0.3% (V : V) acetic acid in water as phase B. The gradient program for chromatographic separation, as indicated in Table 1, was as follows: *T*_min_ A : B): *T*_0_ 20 : 80; *T*_4_ 20 : 80; *T*_20_ 60 : 40; *T*_30_ 60 : 40. Detection was performed at a wavelength of 254 nm, and the flow rate was set as 1.0 mL/min. Additionally, the column temperature was kept constant at 25°C, and the injection volume for all samples was 5 *μ*L.  Figure 4 depicts the chromatogram of QCN (purity >98%), KPL (purity >98%) reference substance, and crude extract A from *Centella asiatica* (L.) Urban, respectively.

#### 2.3.3. ESI-MS^n^ Condition for the Determination of AA, QCN, and KPL

For AA, ESI-MS^n^ was operated with a sheath flow rate of 40 psi, an ion spray voltage of 4.5 kV, and a heated capillary temperature of 350°C. The peristaltic pump flow rate was maintained at 10 *μ*L/min. MS^n^ product ion spectra were generated through collision-induced dissociation (CID) of the deprotonated molecule ion [M-H]^−^ of the analyte at an isolation width (*m/z*) of 1.0. The collision energy for the analyte fell within the range of 25% to 38%. For QCN and KPL, ESI-MS/MS was operated with a sheath flow rate of 20 psi, an ion spray voltage of 4.0 kV, and a heated capillary temperature of 350°C. The peristaltic pump flow rate was also consistently set at 10 *μ*L/min. MS/MS product ion spectra were generated through collision-induced dissociation (CID) of the deprotonated molecule ion [M-H]^−^ of the analyte at an isolation width (*m/z*) of 1.0. The collision energy for the analyte was fixed at 40%.

#### 2.3.4. Preparation of Crude Extract from *Centella asiatica* (L.) Urban

The preparation of *Centella asiatica* (L.) Urban extract was carried out with a slight modification to the previous method established in our laboratory [4]. *Centella asiatica* (L.) Urban was dried to a constant weight at 40°C, crushed, and sifted through a 40-mesh sieve. Ultrasonic-assisted extraction was performed according to the optimized extraction process established by our group, using a solid-liquid ratio of 1 : 15, an ethanol concentration of 75%, and ultrasonic treatment at 40°C for 1 hour. The extracted liquid was filtered on a Brinell funnel with a filter paper, and the residue was extracted twice under the same conditions. The filtrate was combined and reduced pressure concentration was carried out with a rotary evaporator at 40°C. The resulting ethanol extract of *Centella asiatica* (L.) Urban is the *crude extract A*, which is used for the isolation and purification of QCN and KPL. An appropriate amount of crude extract A was extracted using an ethyl acetate-water (2 : 1, v/v) system in a liquid separation funnel, and the ethyl acetate layer was collected. The water layer was extracted twice with twice the volume of ethyl acetate, combined with the ethyl acetate layer, and then decompressed and concentrated using a rotary evaporation instrument at 40°C. The resulting extract was dried in an oven at 40°C to a constant weight (*crude extract B*) and stored in vacuum dry storage for further use (used for the isolation and purification of AA).

#### 2.3.5. Selection of a Solvent System

The optimal solvent system for AA was selected based on the K value of different solvent systems. Three potential solvent systems, namely, *n*-hexane-*n*-butanol-methanol-water (solvent system A), *n*-hexane-*n*-butanol-ethanol-water (solvent system B), and *n*-hexane-*n*-butanol-acetonitrile-water (solvent system C), were selected for further screening based on the average polarity of the solvent system and the physicochemical properties of AA [32]. Ten different solvent systems were prepared according to the conditions listed in Table 2 and screened one by one.

The *K* value was used as an assessment indicator for selecting the two-phase solvent system that was ultimately used for the HSCCC separation [32]. The *K* value of the target components was estimated through HPLC analysis as follows:

The solvent system was mixed in a liquid separation funnel and shaken thoroughly at room temperature. Then, 10.0 mL of upper phase solution and 10.0 mL of lower phase solution were transferred into another liquid separation funnel, and approximately, 20 mg of crude extract B was added. The funnel was plugged and shaken vigorously for about one minute to allow the sample to equilibrate between the two phases. Equal volumes (2.0 mL) of solution from the upper and lower phase solutions were dried out by rotary evaporators at 40°C. The residues were diluted with methanol, and the analytical HPLC system was used to determine the peak area (*A*) of AA in the two phases of the solvent system. The partition coefficient, *K*, was the ratio of AA's peak area *A*_*s*_ in the stationary phase to *A*_*m*_ in the mobile phase, that is, *K* = *A*_*s*_/*A*_*m*_.

Based on the physicochemical properties of QCN and KPL, the classical *n*-hexane-ethyl acetate-methanol-water system was selected as the solvent system to screen these compounds. This solvent system is commonly used for extracting plant secondary metabolites with different polarities. The solvent system screening methods for the separation of QCN and KPL were similar to those used for AA.

#### 2.3.6. Selection of Other Separation Conditions

To ensure the best HSCCC separation, the selection of other separation conditions such as rotation speed and the flow rate of the mobile phase was investigated [32]. Three different rotational speeds (800 rpm/min, 900 rpm/min, and 1000 rpm/min) were tested in countercurrent chromatography experiments to determine their effect on the retention rate and separation efficiency of the stationary phase. Similarly, two different flow rates of the mobile phase (1.5 mL/min and 2.0 mL/min) were tested to determine their impact on the retention rate and separation efficiency of the stationary phase. These experiments were conducted to ensure the optimal conditions for the HSCCC separation process.

#### 2.3.7. Preparation of the Solvent System and Sample Solution

In this study, the selected two-phase solvent system was fully equilibrated in the separation funnel by vigorously shaking it for 2 minutes at room temperature. Prior to use, the two phases were separated and degassed through sonication for 15 minutes. To prepare the sample solutions for HSCCC separation, 200.0 mg of crude extract B was dissolved in 10 mL of the upper phase (solvent system for AA) and 500.0 mg of crude extract A was dissolved in 10 mL of the upper phase (solvent system for QCN and KPL).

#### 2.3.8. HSCCC Separation Procedure

First of all, the semipreparative HPLC system was connected to the TBE 300A HSCCC system. After starting the circulating water bath, the multilayer spiral chromatographic column was completely filled with the upper phase (stationary phase) at a flow rate of 25.0 mL/min. Then, the HSCCC host was started, and the appropriate rotation mode (forward/reverse rotation) was selected. The rotation speed was slowly adjusted to the optimized speed, and the mobile phase was pumped at the optimized flow rate. When a clear mobile phase flowed out from the tail end, it indicated that the two-phase solvent had reached hydrodynamic equilibrium in the instrument, and the sample could be injected through the injection valve at this point. Once the system reached hydrodynamic equilibrium, 10.0 mL of the test solution was injected into the sample valve and eluted in an appropriate elution mode. The effluent was monitored and recorded online at 210 nm (for AA) or 254 nm (for QCN and KPL) using the Waters Empower Workstation of the semipreparative HPLC system. Based on the HSCCC spectrum monitored at 210 nm or 254 nm, the column effluents between the half-peak widths of each peak were continuously collected. After that, the generated fractions were dried at 40°C under reduced pressure and then dried in an oven at 40°C to a constant weight. The powder was then stored in vacuum-dry storage for further use. Once the separation was complete, the solvents in the column were expelled to estimate the retention ratio of the solvent system. The retention ratio (*R*) of the stationary phase was calculated using the equation *R* (%) = (320 − *V*_*L*_)/320, where *R* represents the retention ratio of the stationary phase (upper phase) and *V*_*L*_ is the volume of stationary phase that flowed out of the column [27, 33].

#### 2.3.9. Determination of FAS Enzyme Activity and Inhibitory Activity

The activity of FAS was determined using ultraviolet spectrophotometry, following the method reported by Tian et al. [34]. The procedure is as follows: At 37°C, 1.85 mL of phosphate buffer (pH = 7.0), 25 *μ*L of acetyl-CoA (0.2 mmol/L), 50 *μ*L of malonyl CoA (0.4 mmol/L), 50 *μ*L of NADPH (1.3 mmol/L), and an appropriate amount of blank solvent were added into 2 mL colorimetric dishes successively. Then, 20 *μ*L of FAS solution was added and mixed to start the enzymatic reaction. The activity of FAS enzyme *A*_0_ was determined by UV spectrophotometry at 340 nm.

#### 2.3.10. Determination of FAS Inhibitory Activity

All samples were dissolved in 50% ethanol. Each crude extract (A and B), as well as AA, MA, QCN, and KPL, were prepared in six series of solutions with concentrations of 3.12 *μ*g/mL, 6.25 *μ*g/mL, 12.5 *μ*g/mL, 25.0 *μ*g/mL, 50.0 *μ*g/mL, and 200 *μ*g/mL, respectively. Using the method described in Section 2.3.9, each sample solution was substituted for the blank solvent to measure FAS enzyme activity (*A*_*i*_) at different concentrations. *A*_*i*_/*A*_0_ represents the remaining activity after FAS combines with the sample solution.

#### 2.3.11. Determination of Half-Inhibitory Concentration (IC_50_)

Regression analysis was performed using the SPSS 19.0 software. The Probit analysis logistic model was then used to calculate the IC_50_ value of the sample solution and its corresponding confidence interval, based on the logarithm of the sample mass concentration corresponding to the residual activity value. The IC_50_ value represents the inhibitory capacity of the inhibitor, with a smaller value indicating a stronger inhibitory capacity. Therefore, IC_50_ can be considered an important parameter for evaluating the FAS inhibition activity of the samples.

## 3. Results and Discussion

### 3.1. Optimization of the HSCCC Two-Phase Solvent System

The key factor in achieving a successful HSCCC separation is identifying an appropriate two-phase solvent system. The search for a suitable two-phase solvent system accounts for 90% of the entire work in HSCCC [27, 35]. The selection of the solvent system for HSCCC separation is based on golden rules for selecting optimum conditions for high-speed counter-current chromatography and the difference in partition coefficients (*K*) of the target compound between the two-phase systems [32]. The *K* value in the range of 0.5–2.0 for the target compound is generally considered appropriate for HSCCC separation [31, 35]. As mentioned previously, selecting an appropriate solvent system is one of the most crucial steps in achieving successful HSCCC separation. The partition coefficient (*K*) value is the most significant indicator for determining the resolution in HSCCC. If the *K* value is less than 0.5, the material being separated will be eluted too quickly, resulting in low resolution. Conversely, if the *K* value is greater than 2.0, the peak retention time of the material will be too long, leading to peak broadening and a reduction in separation efficiency.

#### 3.1.1. Selection of a Two‐Phase Solvent System for the Isolation of AA

The partition coefficients (*K* values) of AA in crude extract B were measured in ten different two-phase solvent systems selected from three potential solvent systems using HPLC with UV detection at 210 nm. The results are summarized in Table 3.

According to Table 3, it can be seen that for solvent systems A and B, there exist solvent systems with a distribution coefficient *K* within the range of 0.5–2 as the proportion is adjusted. However, the distribution coefficients *K* of samples in the C1, C2, and C3 systems are all less than 0.5. Additionally, it was found that system C easily forms three layers upon adjusting the proportion, making it unsuitable for this experiment. Then, solvent systems with *K* values within the range of 0.5–2 in Table 3 were selected for the HSCCC fixed phase retention test. However, the results showed that the stationary phase retentions of systems A2, A3, A4, B1, and B2 were all below 30%, failing to meet the requirement of the HSCCC stationary phase retention of more than 40%. Therefore, the above conditions are not desirable.

It is speculated that the low retention rate of the fixed phase may be due to the occurrence of emulsification between the two incompatible phases of the solvent system during the high-speed repeated extraction process. Reducing the emulsification phenomenon can increase the retention rate of the fixed phase. After multiple screenings, it was found that the stationary phase retention could be significantly increased by decreasing the proportion of butanol or increasing the proportion of methanol in the solvent system. Moreover, the solvent system containing ethanol was found to be more prone to emulsification than the solvent system containing methanol.

Based on the above experimental results, system A was further screened to optimize the optimal solvent system. The composition and distribution coefficient *K* of the new solvent system are shown in Table 4.

According to Table 4, both solvent systems *A*_2_ and *A*_4_ meet the requirements for distribution coefficients *K*, and the further stationary phase retention tests indicate that the stationary phase retention *s* of both systems are above 70%. Taking into account the polarity and solubility of the sample, solvent system *A*_2_, composed of *n*-hexane, *n*-butanol, methanol, and water (3 : 1 : 2 : 3, V : V : V : V), was chosen as the experimental condition for HSCCC separation and purification of AA.

#### 3.1.2. Selection of a Two‐Phase Solvent System for the Isolation of QCN and KPL

The partition coefficients (*K* values) of QCN and KPL in crude extract A were measured using HPLC with UV detection at 254 nm. Based on the polarity of QCN and KPL, the classical solvent system of *n*-hexane-ethyl acetate-methanol-water was selected for separation. The proportion of the solvent system was adjusted through the method given in Section 3.4 and repeatedly screened based on the respectively distribution coefficient *K* of QCN and KPL. Finally, the optimal solvent system was determined to be *n*-hexane-ethyl acetate-methanol-water (0.8 : 0.9 : 1.2 : 1, V : V : V : V).

### 3.2. Selection of Other Separation Conditions

Various factors were analyzed, including the rotation speed and the flow rate of the mobile phase. The flow rate of the mobile phase directly affects the amount of stationary phase fixed in the column, separation time, and peak resolution [29, 32]. In this study, the effect of solvent systems at rotation speeds of 800 rpm/min, 900 rpm/min, and 1000 rpm/min on the retention of the target compound's stationary phase was investigated by comparing the retention of AA, QCN, and KPL in HSCCC. Results showed that an increase in rotation speed led to a slight increase in the retention of the stationary phase and the separation effect of AA. However, there was no significant difference in the influence of the solvent systems *n*-hexane, *n*-butanol, methanol, and water (3 : 1 : 3 : 3, V : V : V : V) on the stationary phase retention and separation effect. Since a high rotation speed can cause emulsification between immiscible phases and affect the instrument's service life, a rotation speed of 800 rpm/min was selected for the separation of AA.

Further exploration was conducted on the flow rate of the mobile phase, and the effect of flow rates of 1.5 mL/min and 2.0 mL/min on the separation of AA, QCN, and KPL was examined. The results showed that both flow rates had no significant effect on the separation of AA, QCN, and KPL, but the separation time was longer at a flow rate of 1.5 mL/min. Therefore, a flow rate of 2.0 mL/min was chosen as the flow rate of the mobile phase. Furthermore, a slight increase in the stationary phase retention of QCN and KPL was observed with an increase in rotation speed, but the effect was not significant. However, the separation effect of the sample was significantly improved with an increase in rotation speed. Therefore, a rotation speed of 1000 rpm/min was selected for this experiment. Under the aforementioned conditions, the solvent system used for the separation of AA exhibits a stationary phase retention rate of 70%, whereas the solvent system used for the simultaneous separation of QCN and KPL displays a retention rate of over 65%.

### 3.3. Separation of AA from Crude Extract B by HSCCC

**Figure 5:**
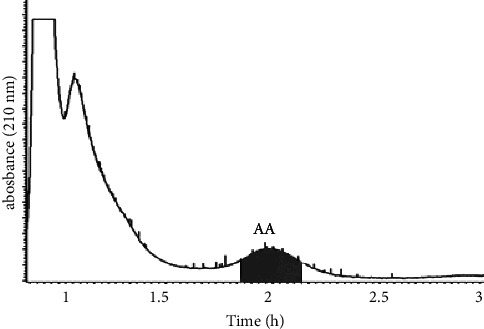
The HSCCC chromatogram of crude extract B at 210 nm.

**Figure 6:**
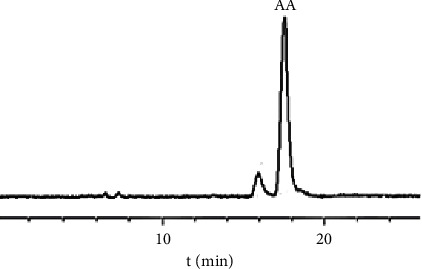
HPLC chromatogram of AA sample purified by HSCCC (210 nm).

To perform the HSCCC separation, 200 mg of crude extract B was dissolved in 10.0 mL of the upper phase of a *n*-hexane-*n*-butanol-methanol-water (3 : 1 : 3 : 3, v/v/v/v) system. The stationary phase used was the upper phase, and the lower phase was used as the mobile phase, with a head-to-tail (forward) elution mode according to the procedure described in Section 2.3.7. The eluent was collected at the half-peak width of the absorption peak in the HSCCC chromatogram and was subsequently filtered and analyzed by HPLC under the conditions described in Section 2.3.1, as shown in Figures 5 and 6.

**Figure 7:**
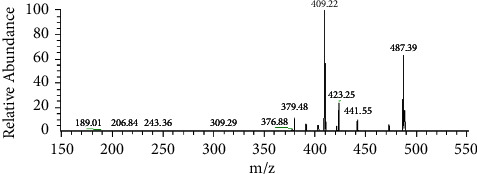
MS/MS spectra of the effluent separated from crude extract B by HSCCC.

Then, ESI-MS^n^ was used for further verification of AA under the conditions described in Section 2.3.3. To ensure an adequate number of fragment ions, a solution containing 10 *μ*g/mL of the effluent separated from crude extract B by HSCCC in methanol was utilized for the fragmentation pattern study. The quasi-molecular ion of the effluent in negative mode exhibited an *m/z* value of 487.39. Its MS/MS spectra and the data of the MS^n^ spectra for its primary fragment ions are presented in Figure 7 and Table 5.

The results demonstrated that the purified sample obtained through HSCCC exhibited a peak shape and retention time in the HPLC chromatography spectrum, UV spectrum, and ESI-MS/MS spectrum, and the data of the ESI-MS^n^ spectrums were consistent with those of the AA reference substance. These findings strongly indicate that the compound is highly likely to be AA. Based on the data presented in Figure 6, the purity of AA was calculated to be 91.0%.

### 3.4. Separation of QCN and KPL from Crude Extract A by HSCCC

**Figure 8:**
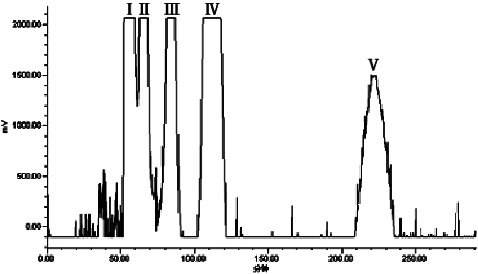
The HSCCC chromatogram of crude extract A at 254 nm.

**Figure 9:**
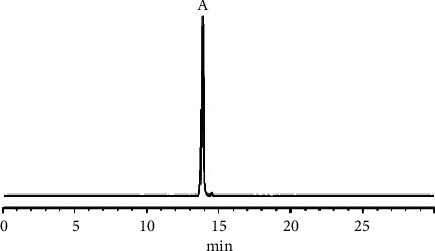
HPLC chromatogram of the distillate IV at 254 nm.

**Figure 10:**
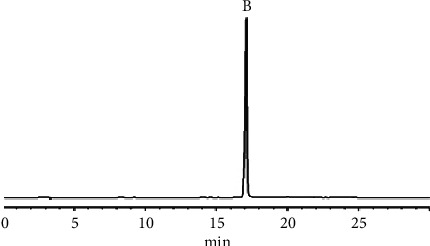
HPLC chromatogram of the distillate V at 254 nm.

To perform the HSCCC separation, 500 mg of crude extract A was dissolved in 10.0 mL of the upper phase of a *n*-hexane-ethyl acetate-methanol-water (0.8 : 0.9 : 1.2 : 1, v/v/v/v) system, and elution was carried out using the upper phase as the stationary phase and the lower phase as the mobile phase in a head-to-tail (forward) manner according to the method described in Section 2.3.7. The HSCCC chromatogram of crude extract A at 254 nm is shown in Figure 8; the eluent was collected from the half-peak width of the absorption peaks IV and V. After filtration through a 0.22 *μ*m filter membrane, the appropriate amount of distillate was taken and analyzed by HPLC under the conditions described in Section 2.3.2, as shown in Figures 9 and 10.

**Figure 11:**
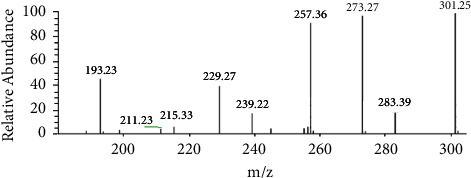
MS/MS spectra of the distillate IV.

**Figure 12:**
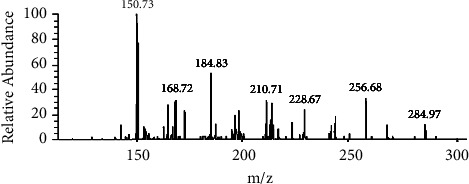
MS/MS spectra of the distillate V.

Then, ESI-MS/MS was employed for the further verification of distillates IV and V, which were separated from crude extract A using HSCCC, under the conditions described in Section 2.3.3. To ensure an adequate number of fragment ions, solutions containing 10 *μ*g/mL of distillates IV and V were respectively prepared in methanol. The quasi-molecular ions of distillates IV and V in negative mode were observed at *m*/*z* 301.25 and *m*/*z* 284.97, respectively. The MS/MS spectra of distillates IV and V in negative mode are presented in Figures 11 and 12, respectively.

The results showed that peaks IV and V in the HSCCC were all single components. Among them, the purified samples corresponding to peaks IV and V had a peak shape, retention time, UV spectrum, and ESI-MS/MS spectrum consistent with those of the QCN and KPL reference substances, respectively, indicating that peak IV was indeed QCN and peak V was indeed KPL. In addition, the HPLC analysis of QCN and KP showed retention times of 14.0 min and 17.0 min, respectively, with purities of 99.1% and 99.2%, respectively. Furthermore, peaks I and II in the HSCCC corresponded to a mixture of multiple components with retention times within 4 min in the HPLC spectrum and did not contain QCN and KPL components. In this study, the HSCCC technique was used to isolate and purify QCN (peak IV) and KPL (peak V) from the crude extract A of *Centella asiatica* (L.), with purities >99% measured by HPLC (as shown in Figures 9 and 10).

**Figure 13:**
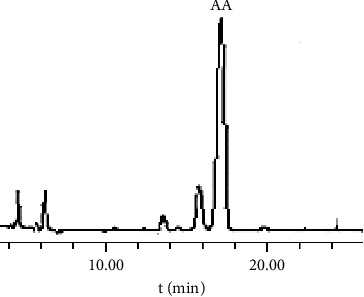
HPLC chromatogram of AA sample purified thrice by column chromatography [26].

In this study, the HSCCC technique was successfully used in isolating and purifying AA, QCN, and KPL with high purity levels. In the previous stage of this study, our team employed silica gel column chromatography with a petroleum ether: acetone system to separate and purify asiatic acid from *Centella asiatica* (L.) Urban [26]. The results showed that a single separation by column chromatography only yielded AA with a purity of 4.6%, and even after repeating the separation by column chromatography three times, the purity was only 79.0% (shown in Figure 13), which is much lower than the purity achieved by HSCCC in this study.

### 3.5. FAS Inhibition Activity of Each Sample from *Centella asiatica* (L.) Urban

**Figure 14:**
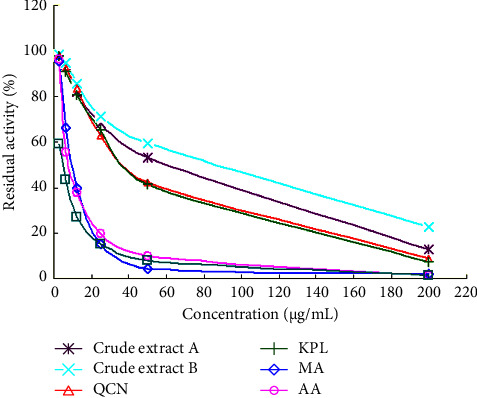
The residual activity of FAS after treating different concentrations of sample solutions extracted from traditional Chinese medicine *Centella asiatica* (L.) Urban (*n* = 3).

This study investigated the inhibitory effects of seven substances extracted from traditional Chinese medicine *Centella asiatica* (L.) Urban on FAS activity at different concentrations. The residual activity value of FAS was used to determine the sample's ability to inhibit FAS. The results are illustrated in Figure 14, indicating a dose-response relationship between the sample concentration and FAS activity inhibition. Of the six substances, AA and MA were found to have stronger inhibitory effects on FAS than crude extracts A and B, QCN, and KPL. Additionally, QCN and KPL exhibited similar inhibitory effects, while MA and AA had similar but stronger effects. The relatively weak inhibitory effects of crude extract A and B were attributed to the presence of inactive impurities. Lastly, the study revealed a positive correlation between dose and effect, with the inhibitory effect of the sample on FAS activity becoming stronger at higher concentrations.

SPSS 19.0 software was used for statistical analysis, and the IC_50_ values of effective components of *Centella asiatica* (L.) Urban FAS inhibition were obtained, as shown in Table 5. The results showed that AA and MA had the strongest inhibitory effect on FAS with IC_50_ values of 9.52 ± 0.76 *μ*g/mL and 10.84 ± 0.92 *μ*g/mL, respectively. MA is the hydroxylation product of AA and is also an important component of *Centella asiatica* (L.) Urban. These results could confirm the existence of a variety of compounds with strong inhibitory effects on FAS activity in *Centella asiatica* (L.) Urban. As shown in Table 6, triterpenoids exhibited stronger FAS inhibition activity compared to flavonoids QCN and KPL, as well as AA and MA.

## 4. Conclusions

The establishment of efficient and effective methods for the isolation of bioactive compounds from natural sources is crucial for their potential use in various applications. This study successfully demonstrated the potential of HSCCC-Semi-Prep -HPLC separation in the rapid and large-scale isolation of AA, QCN, and KPL from *Centella asiatica* (L.) Urban. These methods resulted in high purity (>91.0% for AA and >99.1% for QCN and KPL) and large-scale isolation of the compounds by only one-step HSCCC-Semi-Prep-HPLC separation under the optimized conditions. The study also identified that QCN and KPL had weaker inhibitory effects on FAS compared to AA and MA. Overall, the study provides valuable information for the efficient extraction and separation of bioactive compounds from *Centella asiatica* (L.) Urban and their potential use as inhibitors of FAS.

## Figures and Tables

**Table 1:** Gradient elution procedure for the mobile phase.

Time (min)	A (%)	B (%)
0	20	80
4	20	80
20	60	40
30	60	40

**Table 2:** Screening of the solvent system for AA.

Serial number	Solvent system	Component ratio (V : V : V : V)
1	A-1	1 : 1 : 1 : 1
2	A-2	1 : 1.1 : 1 : 1
3	A-3	1 : 1.1 : 0.9 : 1
4	A-4	1.5 : 1 : 0.9 : 1
5	A-5	1.5 : 1 : 0.8 : 1
6	B-1	1.1 : 1 : 1.1 : 1
7	B-2	1.5 : 1 : 0.8 : 1
8	C-1	1 : 1 : 1 : 1
9	C-2	1.5 : 1 : 0.9 : 1
10	C-3	1.8 : 1 : 0.8 : 1

Solvent system A: *n*-hexane-*n*-butanol-methanol-water. Solvent system B: *n*-hexane-*n*-butanol-ethanol-water. Solvent system C: *n*-hexane-*n*-butanol-acetonitrile-water.

**Table 3:** Distribution coefficient of AA in 10 different solvent systems.

Serial number	Solvent system	Component ratio (V : V : V : V)	*K* value
1	A-1	1 : 1 : 1 : 1	0.49
2	A-2	1 : 1.1 : 1 : 1	0.61
3	A-3	1 : 1.1 : 0.9 : 1	0.96
4	A-4	1.5 : 1 : 0.9 : 1	0.75
5	A-5	1.5 : 1 : 0.8 : 1	0.11
6	B-1	1.1 : 1 : 1.1 : 1	0.94
7	B-2	1.5 : 1 : 0.8 : 1	1.01
8	C-1	1 : 1 : 1 : 1	0.25
9	C-2	1.5 : 1 : 0.9 : 1	0.43
10	C-3	1.8 : 1 : 0.8 : 1	0.27

Solvent system A: *n-*hexane-*n-*butanol-methanol-water. Solvent system B: *n-*hexane-*n-*butanol-ethanol-water. Solvent system C: *n-*hexane-*n-*butanol-acetonitrile-water.

**Table 4:** The partition coefficient *K* of AA in solvent system A.

Serial number	Component ratio (V : V : V : V)	*K* value
*A *_1_	2 : 1 : 2 : 2	0.48
*A *_2_	3 : 1 : 3 : 3	1.02
*A *_3_	3 : 2 : 3 : 2	0.17
*A *_4_	3 : 1 : 2 : 3	1.56

Solvent system A: *n-*hexane-*n*-butanol-methanol-water.

**Table 5:** ESI-MS^n^ negative ions of the effluent separated from crude extract B by HSCCC.

[M-H]^−^	Scan mode (*m/z*)	Main fragmentation ions (*m/z*)
487	MS^2^ (487)	487, 473, 441, 423, 409, 391, 379
	MS^3^ (487⟶441)	421, 409, 379, 233
	MS^3^ (487⟶423)	405, 393, 347
	MS^3^ (487⟶409)	391, 379, 375

**Table 6:** IC_50_ value of each ingredient extracted from *Centella asiatica* (L.) Urban in the inhibition of FAS (*n* = 3).

No.	Sample solution	IC_50_ (*μ*g/mL)
1	Crude extract A	48.15 ± 2.51
2	Crude extract B	57.02 ± 3.59
3	QCN	43.09 ± 2.98
4	KPL	36.90 ± 1.83
5	AA	9.52 ± 0.76
6	MA	10.84 ± 0.92

## Data Availability

The data that support the findings of this study are available from the first author, Binbin Xia, and corresponding author, Hua Cheng, upon reasonable request.
